# Sequestration of Highly Expressed mRNAs in Cytoplasmic Granules, P-Bodies, and Stress Granules Enhances Cell Viability

**DOI:** 10.1371/journal.pgen.1002527

**Published:** 2012-02-23

**Authors:** Anna Lavut, Dina Raveh

**Affiliations:** Department of Life Sciences, Ben Gurion University of the Negev, Beersheba, Israel; Stanford University School of Medicine, United States of America

## Abstract

Transcriptome analyses indicate that a core 10%–15% of the yeast genome is modulated by a variety of different stresses. However, not all the induced genes undergo translation, and null mutants of many induced genes do not show elevated sensitivity to the particular stress. Elucidation of the RNA lifecycle reveals accumulation of non-translating mRNAs in cytoplasmic granules, P-bodies, and stress granules for future regulation. P-bodies contain enzymes for mRNA degradation; under stress conditions mRNAs may be transferred to stress granules for storage and return to translation. Protein degradation by the ubiquitin-proteasome system is elevated by stress; and here we analyzed the steady state levels, decay, and subcellular localization of the mRNA of the gene encoding the F-box protein, *UFO1*, that is induced by stress. Using the MS2L mRNA reporter system *UFO1* mRNA was observed in granules that colocalized with P-bodies and stress granules. These P-bodies stored diverse mRNAs. Granules of two mRNAs transported prior to translation, *ASH1-MS2L* and *OXA1-MS2L*, docked with P-bodies. *HSP12* mRNA that gave rise to highly elevated protein levels was not observed in granules under these stress conditions. *ecd3*, *pat1* double mutants that are defective in P-body formation were sensitive to mRNAs expressed ectopically from strong promoters. These highly expressed mRNAs showed elevated translation compared with wild-type cells, and the viability of the mutants was strongly reduced. *ecd3, pat1* mutants also exhibited increased sensitivity to different stresses. Our interpretation is that sequestration of highly expressed mRNAs in P-bodies is essential for viability. Storage of mRNAs for future regulation may contribute to the discrepancy between the steady state levels of many stress-induced mRNAs and their proteins. Sorting of mRNAs for future translation or decay by individual cells could generate potentially different phenotypes in a genetically identical population and enhance its ability to withstand stress.

## Author Summary

10%–15% of the yeast genome is modulated by stress; however, there is a discrepancy between the genes that are upregulated and the sensitivity of the null mutants of those genes to the stress. The question is: what happens to these highly expressed mRNAs? mRNAs have a complex lifecycle and non-translating mRNAs can be stored in cytoplasmic granules, processing P-bodies, and stress granules for decay or future translation, respectively. *UFO1* encodes a component of the regulated protein degradation system, and its transcription is elevated by stress; however, the deletion mutants do not show enhanced sensitivity. *UFO1* mRNA is stored in P-bodies and stress granules. Storage of mRNAs may contribute to the discrepancy between the steady state levels of stress-induced mRNAs and their proteins. To test this hypothesis, we expressed high levels of mRNA in cells unable to form P-bodies. We found that translation of these mRNAs was 3–8 fold higher than in wild-type cells. Furthermore high level expression of mRNA affected the viability of the mutants. The ability to store mRNAs for future translation or decay would generate different phenotypes in a genetically identical population and enhance its ability to withstand stress.

## Introduction

High throughput yeast microarray studies indicate that the mRNA abundance of a common core of 10–15% of the yeast genome is modulated by a variety of different environmental challenges such as DNA damage, heat, oxidative, osmotic, heavy metal, and salt stress [1]. This response, known as the environmental stress response (ESR) represents a network of interlinked functions that preserves cell integrity [2], [3]. A hallmark of the ESR is a down-regulation of protein synthesis genes and an up-regulation of genes that encode chaperones and genes involved in protein degradation [4].

The ubiquitin-proteasome system is the major pathway for regulated protein degradation in the cell. In most cases proteins targeted to the proteasome are covalently linked to chains of ubiquitin by a cascade of E1, ubiquitin activating- and E2 ubiquitin conjugating enzymes, and an E3 ubiquitin ligase [5]. The large family of Skp1-Cdc53-F-box protein (SCF) ubiquitin ligase complexes regulates growth and cell cycle progression in all eukaryotes [6]. In yeast about seventeen different F-box proteins recruit degradation substrates to the SCF complex. Of all the F-box proteins, transcription only of *UFO1* is highly induced by DNA damage and arsenate stress (four- and sixfold respectively [7], [8]. *UFO1* may function in maintenance of genome stability as in the absence of Pif1 helicase *ufo1Δ* mutants show a 74-fold increase in gross chromosomal rearrangements [9]. This role is consistent with the key function of SCF^Ufo1^ in degradation of the mating switch Ho endonuclease [10]–[13], and the translesion DNA polymerase, Rad30 [14]. However, despite the robust induction of transcription of *UFO1* mRNA in response to stress, *ufo1Δ* mutants do not display enhanced sensitivity to arsenate [8] or UV [15] compared with their isogenic wild types. Functional profiling in yeast shows that this is a widespread phenomenon as deletion mutants of many genes highly induced by a particular stress do not exhibit enhanced sensitivity to the specific stress (reviewed by [16]).

The dynamics of the transcriptome in response to changing conditions is mainly determined by the balance between transcription and mRNA decay and in many instances functionally related genes show a negative correlation between transcription and decay [17], [18]. Genome-wide proteomics revealed that only ca. 70% of the steady state protein levels can be attributed to mRNA abundance, indicative of translational regulation [19]–[23]. Furthermore the lifecycle of mRNA molecules is complex and involves dynamic changes in subcellular localization to distinct cytoplasmic bodies. These include processing bodies (PBs) that are rich in mRNA decay enzymes such as mRNA decapping enzymes (the Dcp1/Dcp2 heterodimer and its activator, Dhh1), Xrn1 5′-3′ exonuclease, and repressors of translation [24]–[27]. The protein composition of PBs suggests that they are centers of mRNA degradation [28]–[30]. Under stress conditions mRNAs are also present in Stress Granules (SGs) that form when initiation of translation is impaired. SGs contain primarily translation initiation factors and may therefore serve as storage centers from which the mRNAs can be returned to the polysomes for translation [27], [29], [31]–[34]. Despite their apparently distinct roles several lines of evidence suggest a functional relationship between PBs and SGs. First, genetic studies show that formation of yeast SGs depends on biogenesis of PBs [27]. Second, PBs and SGs are often colocalized suggesting that mRNAs may be transferred from one body to the other [27]. Third, yeast deficient for the decapping activators, Pat1 and Dhh1, are blocked in both repression of translation and in PB formation [30], [35]. Finally, recent studies link transcription with both mRNA nuclear export, decay, and translation: two subunits of the RNA Pol II holoenzyme transcription complex, Rpb4 and Rpb7, escort the mRNA transcripts from the nucleus to the cytoplasm where they physically interact with subunits of the PBs [36], [37]; these subunits are important for coordination of mRNA synthesis with decay [18]. Additional PB-associated proteins, yeast Dhh1 and its Xenopus ortholog, shuttle between the nucleus and cytoplasm [38], [39]. Thus the discrepancy between transcript and protein level could be attributable to the complex regulatory mechanisms of the mRNA lifecycle.

Here we report analysis of the lifecycle of *UFO1* mRNA whose transcription is highly induced by several kinds of stress, yet the null mutant shows no enhanced sensitivity to these stresses. We compared *UFO1* mRNA lifecycle with that of the heat shock gene, *HSP12* that represents a different paradigm of regulation. *HSP12* is regulated by the ESR and encodes a membrane protein important for preserving membrane organization under stress conditions [40]. Induction of *HSP12* mRNA is slow [41], but the protein attains a high cellular level after stress. We measured induction of *UFO1* and *HSP12* mRNAs and the stability of their mRNAs after stress by quantitative real time PCR (qRT-PCR). We also compared their steady state protein levels in response to different stresses. Furthermore to dissect the *UFO1* mRNA lifecycle we followed the mRNA molecules in single cells by tagging genomic *UFO1* with bacteriophage MS2L sequence for detection with the viral capsid protein fused to GFP [42], [43]. As further reference mRNAs we used *MFA2* mRNA that is constitutively expressed at a high level from a strong promoter [24], [44], the low copy mRNAs of *ASH1* and *OXA1* that are localized to the bud [42] and the mitochondrion [43], respectively, prior to their translation. We visualized the subcellular localization of these mRNA molecules in single cells that expressed fluorescent subunits of the PBs and SGs [27], [31], [45] and found that the highly expressed *UFO1* and *MFA2* mRNAs enter PBs. Genetic analysis using mutants unable to form PBs showed that cell viability is strongly reduced when *UFO1*, *MFA2* or *HSP12* genes are expressed at a high level from the *GAL* promoter. Our interpretation is that sequestration in PBs plays a major role in preserving cell viability. We suggest that the ability to store highly abundant mRNAs in PBs for future regulation is a key facet of the stress response allowing individual cells to sort mRNAs for decay or translation. This mechanism has the potential to facilitate acquisition of a variety of different phenotypes in a genetically identical population enhancing its ability to withstand the stress.

## Results

### Expression of *UFO1* mRNA in cell populations in response to stress

**Figure 1:**
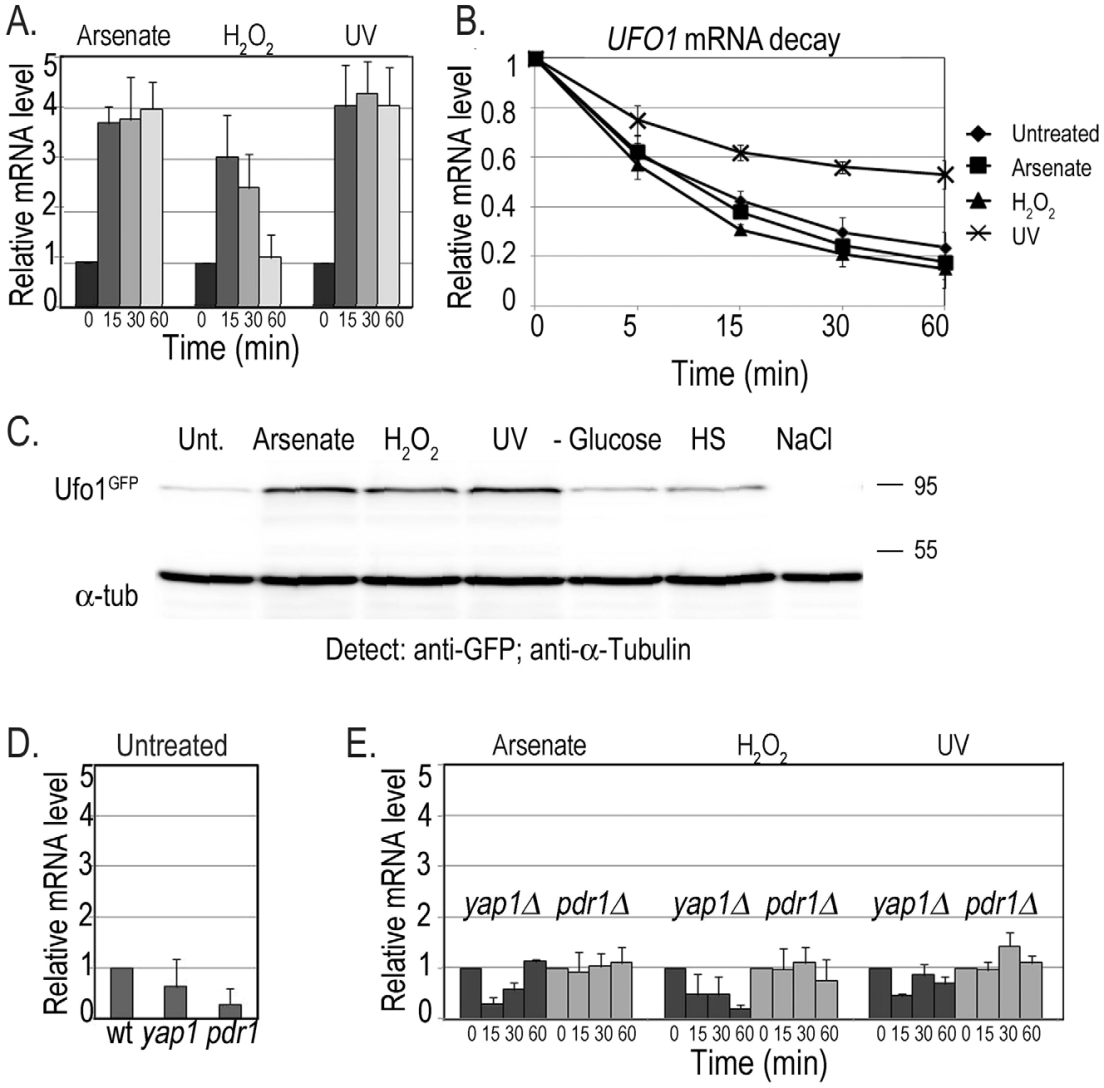
Transcription of *UFO1* in response to arsenate, H_2_O_2_, and UV. A. Wild type cells were grown in SC 2% glucose medium overnight, diluted to *A*_600_ = 0.1, regrown to *A*_600_ = 0.5 and treated with 1 mM arsenate, 8.8 mM H_2_O_2_, or irradiated with 40 mJ/cm^2^ UV. Aliquots were collected at the indicated times and analyzed by qRT-PCR. mRNA levels were normalized to *ACT1* and to time 0 (untreated log cells). B. p*GAL-GFP-UFO1* was expressed in *ufo1Δ* mutants by overnight induction with 2% galactose. Next morning cells were diluted to *A*_600_ = 0.1, regrown to *A*_600_ = 0.5 then untreated, or stressed with 1 mM arsenate or 8.8 mM H_2_O_2_ for 30 minutes, or irradiated with 40 mJ/cm^2^ UV. The cells were washed and transferred to SC with 4% glucose. Samples were collected immediately after addition of glucose and at the times indicated and analyzed by qRT-PCR. mRNA levels were normalized to *ACT1* and to time 0 (untreated log cells). C. Western blot analysis of Ufo1^GFP^ protein produced from the tagged genomic *UFO1-GFP* gene. Anti-GFP antibodies were used to detect Ufo1^GFP^ and anti-α-tubulin antibodies to detect α-tubulin that serves as a loading control. D. *UFO1* mRNA levels in untreated wild type, *yap1Δ* or *pdr1Δ* mutants. mRNA levels were normalized to *ACT1* and to w.t. mRNA levels. E. *yap1Δ* or *pdr1Δ* mutants grown, treated and analyzed as in Figure 1A. mRNA levels were normalized to *ACT1* and to time 0 (untreated log cells).

We followed *UFO1* expression using qRT-PCR on mRNA extracted from cells at different times after treatment with arsenate, H_2_O_2_, or UV irradiation. *UFO1* is under repression during normal growth conditions [46] and treatment with arsenate led to a fourfold increase in the level of *UFO1* mRNA after 15 minutes that stayed high for at least one hour during which the cells were assayed. Treatment with H_2_O_2_ gave a threefold increase in mRNA level after 15 minutes followed by a decrease back to the basal level of the untreated control from the 30 minute time point. After UV irradiation the *UFO1* transcript level increased fourfold after 15 minutes and remained high for one hour (Figure 1A). The mRNA abundance is dependent upon the relative rates of transcription and decay, and when we examined the decay of *UFO1* mRNA in response to the above stresses we observed stabilization of *UFO1* mRNA in response to UV. The half-life of *UFO1* mRNA was ca. 7 minutes in untreated cells and in cells treated with arsenate or H_2_O_2_, but extended to almost 30 minutes after UV irradiation (Figure 1B). Ufo1 protein levels are very low in untreated cells [47], however, by using 10-fold the amount of cells we could observe the protein after arsenate, H_2_O_2_, or UV stress (Figure 1C). ^GFP^Ufo1 protein was stabilized in cells treated with arsenate or H_2_O_2_, but has the same half-life after UV irradiation as in the untreated control cells (Figure S1). Thus the Ufo1 protein could reflect accumulation during the prolonged arsenate or H_2_O_2_ treatments, but in contrast in UV-irradiated cells could be newly translated from the stabilized *UFO1* mRNA. The *UFO1* promoter has sequence elements similar to those regulated by the Yap1 oxidative stress- and Pdr1 pleiotropic drug response transcription factors. Untreated mutant cells showed a reduced basal level of *UFO1* transcription in *yap1Δ* and *pdr1Δ* mutants compared with wild type cells (Figure 1D) and there was no induction of *UFO1* transcription in response to arsenate, H_2_O_2_, and UV stresses indicating that induction of *UFO1* mRNA is Yap1- and Pdr1-dependent (Figure 1E).

### Detection of *UFO1* mRNA in single cells in response to stress

**Figure 2:**
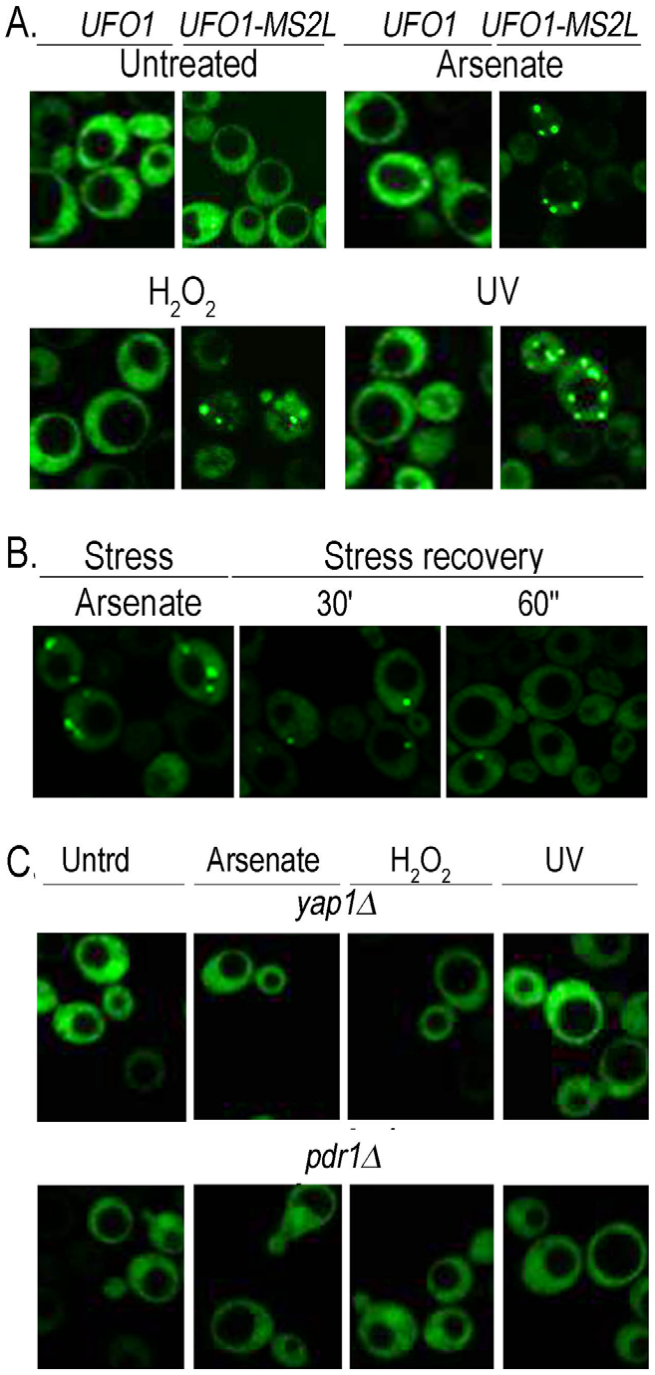
Stress-induced granules appear only in *UFO1-MS2L* stressed cells. A. Wild type and *UFO1-MS2L* cells transformed with p*CP-MS2L-GFPx3* at *A*_600_ = 0.5, were transferred to SC 2% glucose without methionine for 1 hour to induce the CP^GFP^. Cells were untreated or exposed to 1 mM arsenate, 8.8 mM H_2_O_2_, or UV-irradiated with 40 mJ/cm^2^. Aliquots were collected 30 minutes after each treatment. Fluorescent granules appear only in cells with the tagged *UFO1-MS2L* gene. B. *UFO1-MS2L* cells expressing p*CP-MS2L-GFPx3* grown as above, treated with 1 mM arsenate for 30 minutes (stress), and transferred to fresh SC glucose medium. Stress recovery 30′ and 60′ indicate time after transfer to fresh SC. C. *UFO1-MS2L*, *yap1Δ* or *UFO1-MS2L*, *pdr1Δ* cells grown as above and untreated (Utrd), exposed to 1 mM arsenate, 8.8 mM H_2_O_2_, or UV-irradiated with 40 mJ/cm^2^.

Within a population of cells of identical genotype the response of individual cells to stress can differ [48], [49]. Therefore to test the response of single cells we tagged the *UFO1* ORF with *MS2L* DNA and expressed the coat protein (CP) that binds the *MS2L* RNA as a GFP-fusion protein [43]. To ensure the specificity of detection of *UFO1* mRNA by the CP^GFP^ fusion protein we induced CP expression in logarithmic cells with SC glucose medium lacking methionine for one hour. Subsequently, both wild type control and *UFO1-MS2L* cells were treated with arsenate, H_2_O_2_, or UV irradiation and observed after 30 minutes by confocal microscopy. In the control cells the CP^GFP^ protein was visible diffusely throughout the cytoplasm both before and after each stress treatment whereas in response to arsenate, H_2_O_2_, and UV the *UFO1-MS2L* cells showed pronounced cytoplasmic granules ranging in number from zero to five (Figure 2A). Cessation of the treatment by incubation in fresh medium led to their disappearance (Figure 2B). No granules were observed after 30 minutes in treated *UFO1*-*MS2L*, *yap1Δ* or *UFO1*-*MS2L*, *pdr1Δ* mutant cells (Figure 2C) similar to the qRT-PCR results (Figure 1B).

### Time course of granule appearance after stress

**Figure 3:**
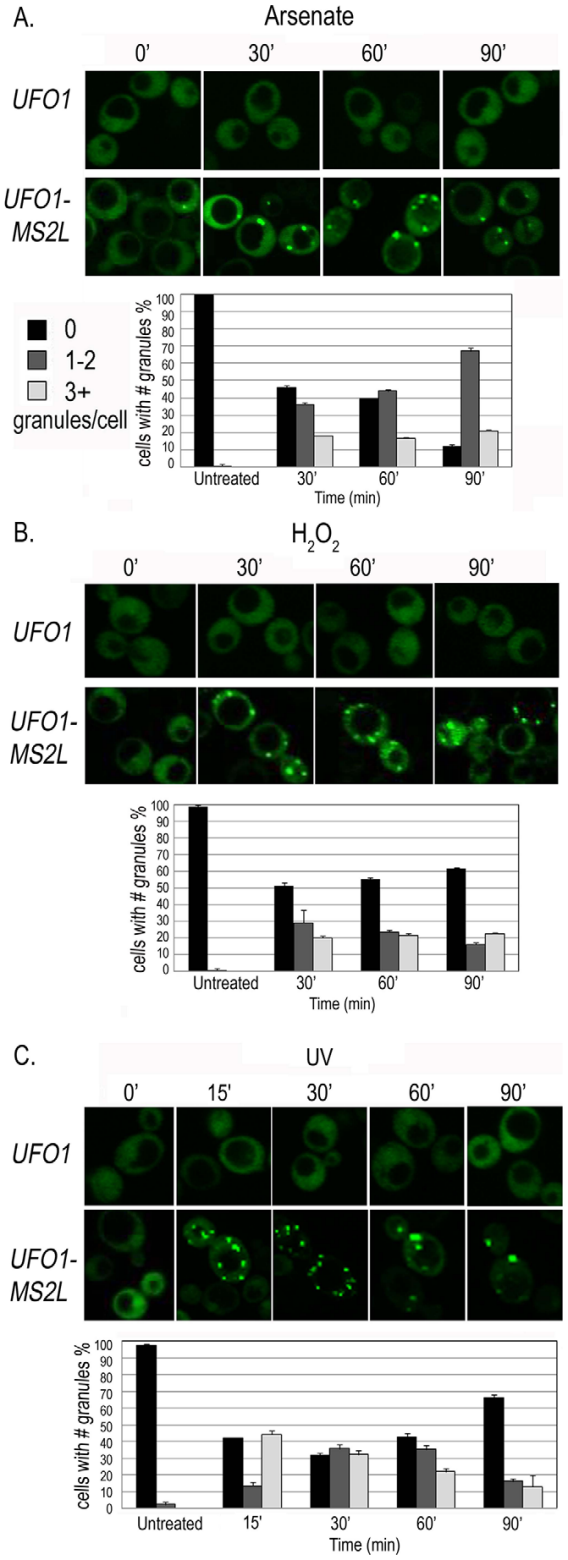
Time course of granule appearance in *UFO1-MS2L* cells after stress. Cells treated with A. 1 mM arsenate, B. 8.8 mM H_2_O_2_, or C. 40 mJ/cm^2^ UV, and visualized by confocal microscopy at the times indicated. Histograms show number of cells with different numbers of granules per cell at the times indicated (n = >150 cells).

Accumulation of the granules was gradual and not all the cells in the population responded within the same time frame and to the same extent. We therefore quantified 150–200 cells for each treatment by defining three different classes of granules per cell: zero, 1–2 (few), or >3 (multiple). In response to arsenate after 30 minutes we observed an increase in the number of cells with a few or multiple granules followed by an increase in cells with a few granules over the 90-minutes of the experiment (Figure 3A). H_2_O_2_ led to an increase in cells with a few and with multiple granules after 30 minutes. By 90 minutes there was a slight increase in cells without granules and a slight decrease in the number of cells with 1–2 granules; the relative number of cells with multiple granules was unaltered (Figure 3B). After UV-irradiation *UFO1-MS2L*-CP^GFP^ granules were visible after15 minutes; this included cells with a few granules, but mostly with multiple granules. The relative number of cells with a few granules increased during the first hour and then decreased; the number of cells with multiple granules decreased from 30 minutes post-irradiation until the end of the experiment (Figure 3C).

### Characterization of the *UFO1* mRNA granules

**Figure 4:**
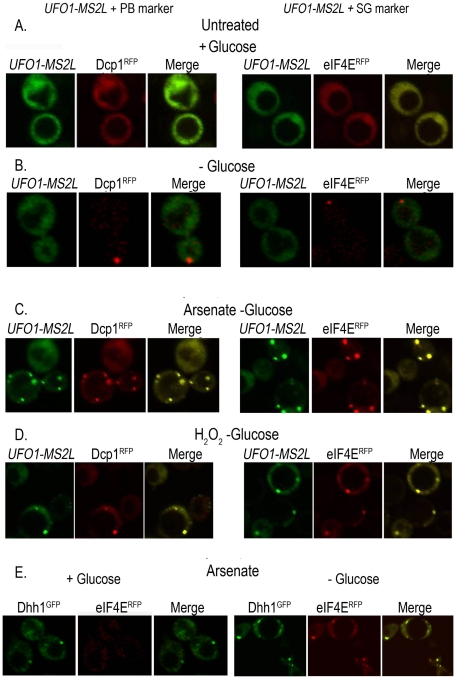
*UFO1-MS2L* mRNAs induced by stress colocalize with subunits of PBs and SGs. A. *UFO1-MS2L* cells at *A*_600_ = 0.5 that produce PB marker protein, Dcp1^RFP^, or the SG marker, eIF4E^RFP^, were transferred to fresh SC 2% glucose medium without methionine for 1 hour. B. Cells as in A, deprived for glucose for 30 minutes. C. Cells as in B, treated for 30 minutes with 1 mM arsenate, or D. 8.8 mM H_2_O_2_. E. Cells at *A*_600_ = 0.5 that produce both the PB marker protein, Dhh1^GFP^, and eIF4E^RFP^, treated with 1 mM arsenate for 30 minutes in the presence or the absence of glucose.

To determine whether the granules that appeared after stress treatment of *UFO1*-*MS2L* cells correspond to PBs or SGs we tested for colocalization of the *UFO1* mRNA with the PB marker protein, Dcp1^RFP^, and with the SG marker protein, eIF4E^RFP^, by mating *UFO1-MS2L* cells with PB- or SG-tagged strains [31]. Neither *UFO1-MS2L* granules nor PBs or SGs were visible in untreated cells growing in glucose medium (Figure 4A); cells in SC medium without glucose showed a single red fluorescent PB or SG, however, there was no induction of *UFO1* mRNA in response to glucose deprivation (Figure 4B) and [31]. After 30 minutes of arsenate or H_2_O_2_ treatment of the glucose-deprived cells, we observed induction of *UFO1* mRNA granules and these colocalized with both the PB and SG marker proteins (Figure 4C and 4D, respectively). Indeed control cells that coexpressed the PB marker protein, Dhh1^GFP^, and the SG marker protein, eIF4E^RFP^, showed overlap between the two types of granules after arsenate treatment (Figure 4E).

### Two distinct highly abundant mRNAs, *UFO1* and *MFA2*, colocalize to the same PBs

**Figure 5:**
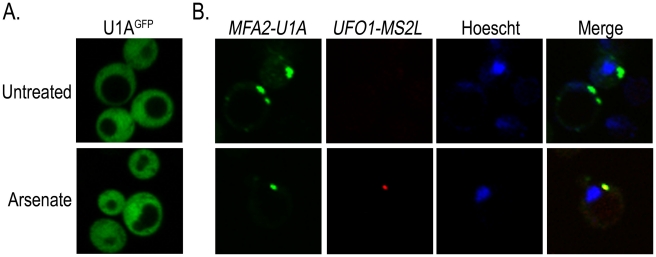
*UFO1-MS2L* and *MFA2-U1A* mRNAs are sequestered in the same PBs after arsenate stress. A. Control wild type cells at *A*_600_ = 0.5 producing U1A^GFP^ untreated or treated with 1 mM arsenate for 30 minutes. B. wild type *UFO1-MS2L* cells at *A*_600_ = 0.5 expressing p*MFA2-U1A*, with their respective RNA-binding proteins, CP^mCherry^ and U1A^GFP^, untreated or treated with 1 mM arsenate and stained with Hoechst 33342 at a final concentration of 2.5 µg/mL for 30 minutes.

The colocalization of stress-induced *UFO1* mRNA with proteins associated with PBs and SGs together with the overlap of these bodies with one another after stress (Figure 4) suggest that these granules may house highly expressed mRNAs. To determine whether more than one mRNA species is present in the same granule we used a second well-characterized mRNA tagging system in which U1A binding sites are inserted into the 3′-UTR of the mRNA of interest and coexpressed with the U1A^GFP^ RNA-binding protein [44]. Wild type cells were transformed with the plasmid for producing U1A^GFP^ protein alone, or cotransformed with p*MFA2-U1A* and the above plasmid. Expression of *MFA2* is regulated by the strong constitutive *GPD* (glyceraldehyde-3-phosphate dehydrogenase) promoter [24], [44] and reaches a high steady state level. As both the U1A and the CP proteins are fused to GFP, we changed the marker of the CP^GFP^ protein to mCherry so that we could detect each mRNA species separately in the same cells. Control untreated or arsenate stressed cells that expressed only U1A^GFP^ protein showed no granule formation. Untreated cotransformants of p*MFA2-U1A* and *UFO1-MS2L* showed multiple *MFA2-U1A* granules, but no granules attributable to *UFO1-MS2L* mRNA. After arsenate treatment *UFO1-MS2L* mRNA granules bound by red CP^mCherry^ were observed and these colocalized with the *MFA2-U1A* granules. This result indicates that multiple mRNA species are found in the same PB (Figure 5).

### Low-abundance mRNAs *ASH1-MS2L* and *OXA1-MS2L* partially colocalize with PBs and SGs

**Figure 6:**
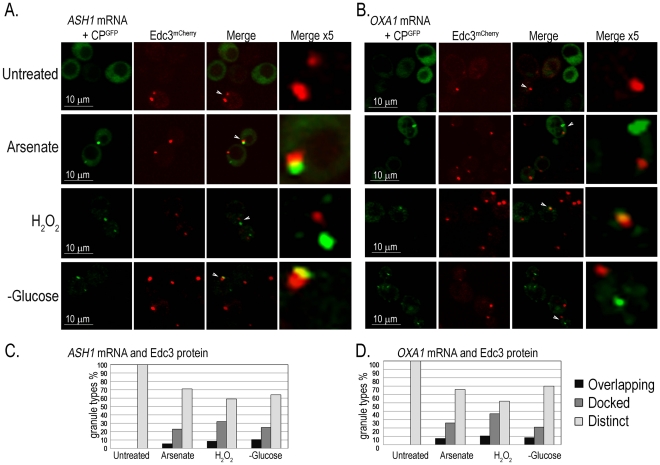
*ASH1* and *OXA1* mRNA granules interact with PBs. A. *ASH1-MS2L* cells at *A*_600_ = 0.5 with p*CP-MS2L-GFPx3* and the PB marker, Edc3^mCherry^, either untreated, treated with 1 mM arsenate or with 8.8 mM H_2_O_2_ for 30 minutes or transferred to SC without glucose for 30 minutes. Merge ×5 represents 5 times enlargement of selected granules indicated with white arrows in the whole cells. B. *OXA1-MS2L* cells treated as in A., and visualized by confocal microscopy. C. Histograms of *ASH1-MS2L* cells or D. *OXA1-MS2L* cells, showing percentages of overlapping, docked, or distinct granule types in a population of cells untreated, treated with 1 mM arsenate or with 8.8 mM H_2_O_2_ for 30 minutes or stressed in SC without glucose (n = >100 cells).

To determine whether all mRNA granules correspond to PBs we assayed two further mRNAs that are specifically localized prior to their translation: *ASH1-MS2L* mRNA that is transported from the mother cell to the bud [42], and *OXA1-MS2L* mRNA that is localized to the mitochondria [43]. The PB marker Edc3^mCherry^ was produced in cells that expressed *ASH1-MS2L* or *OXA1-MS2L* together with p*CP-MS2L-GFPx3* for detection of their mRNAs. The cells were treated with the above stresses and imaged after 30 minutes with the confocal microscope. Both *ASH1-MS2L* and *OXA1-MS2L* mRNAs showed a similar response to the stress treatments. A small number of untreated cells showed at most a few Edc3^mCherry^-marked PBs, however, most cells lacked CP^GFP^ granules of either *ASH1-MS2L* or *OXA1-MS2L* mRNAs. After 30 minutes with arsenate, H_2_O_2_, or glucose deprivation, we observed CP^GFP^ granules corresponding to *ASH1-MS2L* (Figure 6A) or *OXA1-MS2L* (Figure 6B) mRNAs. These granules did not fully colocalize with PBs as did *UFO1* mRNA, however, they were often docked, partially colocalized (overlapping), or appeared in the same cells with the PB marker protein Edc3^mCherry^, but at a distinct location in the same cell (Figure 6C).

### Highly expressed Hsp12 protein is encoded by a mRNA not observed in mRNA granules

**Figure 7:**
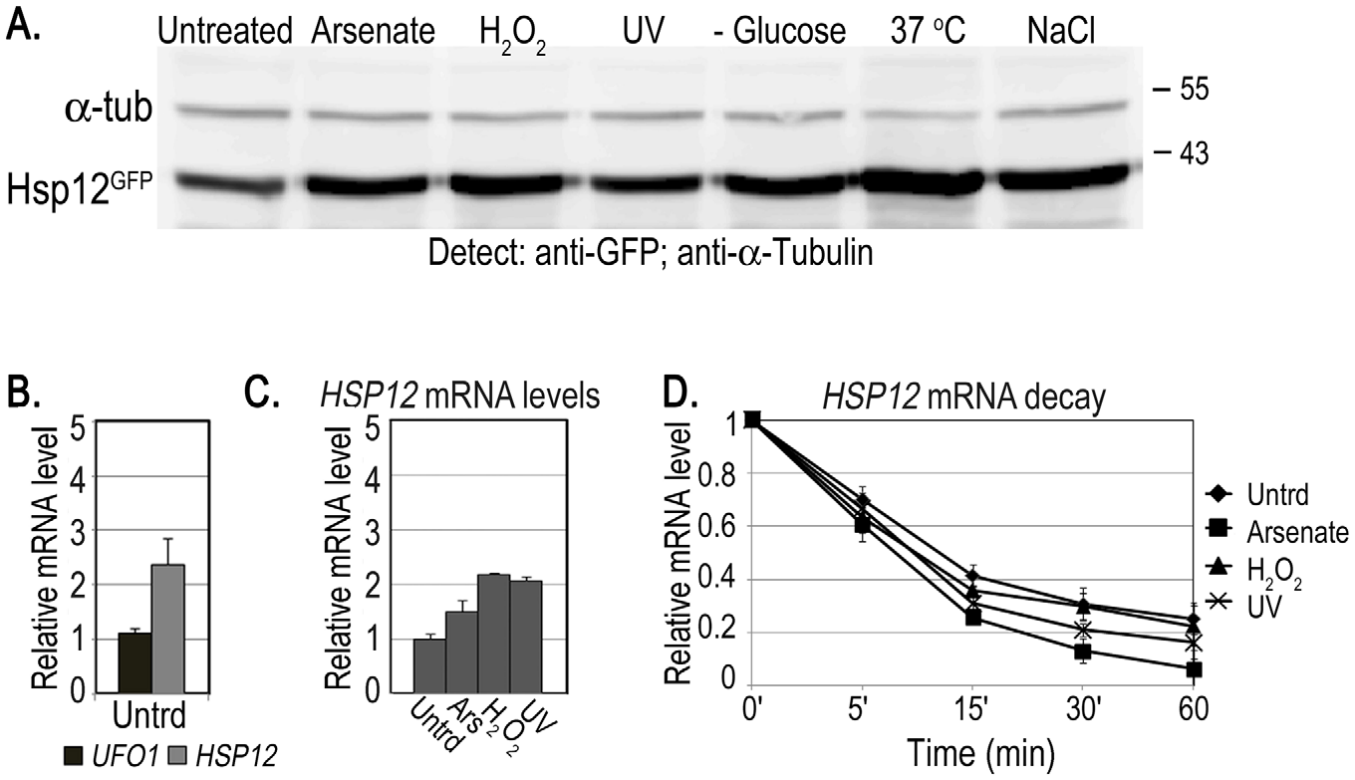
Induction of Hsp12 protein and mRNA, and mRNA decay after stress. A. WB of protein produced from genomic *HSP12-GFP* in response to stress. B. *UFO1* and *HSP12* mRNA levels in untreated cells analyzed by qRT-PCR. mRNA levels were normalized to *ACT1*. C. Induction of *HSP12* mRNA by stress. Wild type cells at *A*_600_ = 0.5, treated with 1 mM arsenate, 8.8 mM H_2_O_2_, irradiated with 40 mJ/cm^2^ UV or shifted from 30°C to 37°C for 40 minutes. Aliquots were collected at the times indicated and analyzed by qRT-PCR. D. *HSP12* mRNA decay. p*GAL-HSP12* was expressed in *hsp12Δ* mutants by overnight induction with 2% galactose. Next morning cells at *A*_600_ = 0.5 were untreated, or stressed with 1 mM arsenate or 8.8 mM H_2_O_2_ for 30 minutes, or irradiated with 40 mJ/cm^2^ UV. The cells were washed and transferred to SC medium with 4% glucose. Samples were collected immediately after addition of glucose and at the times indicated and analyzed by qRT-PCR. mRNA levels were normalized to *ACT1* and to time 0 (untreated log cells).

Both whole-genome microarray experiments [7], [8] and our qRT-PCR data (Figure 1) indicate that *UFO1* steady state mRNA levels are elevated in response to stress. However, it is only by using 10-fold the amount of cells compared with our standard protocols that we are able to detect Ufo1^GFP^ protein after any of the stresses applied. We therefore examined the fate of the mRNA of *HSP12*, the protein of which is highly expressed in response to stress [40]. We treated cells with arsenate, H_2_O_2_, UV, glucose deprivation, 37°C or NaCl and analyzed induction of the protein by Western blotting. Hsp12^GFP^ protein was induced after all the treatments, particularly after 37°C (Figure 7A and Figure S2). To examine mRNA localization we fused the *MS2L* tag to the *HSP12* ORF and treated the cells with the same stresses. In contrast to *UFO1*-*MS2L*, we did not detect granules in *HSP12-MS2L* cells and the CP^GFP^ signal remained diffuse throughout the cytoplasm (Figure S3 and [50]). The basal level of *HSP12* mRNA in untreated cells was higher than that of *UFO1* (Figure 7B). however, the relative increase of *HSP12* steady state mRNA level after stress was lower than the high induced levels of *UFO1* mRNA in these samples (cf. Figure 7C and Figure 1A). The elevation of *HSP12* mRNA level results from increased transcription as *HSP12* mRNA is slightly destablized after arsenate, whereas after H_2_O_2_, and UV treatments the stability is similar to the untreated cells with a half-life of ca. 10 minutes in both untreated and stressed cells (Figure 7D). There was no stabilization of the *HSP12* mRNA after UV irradiation as observed for *UFO1* mRNA (Figure 1B).

### High-level expression of mRNA affects the viability of mutants unable to form PBs

**Figure 8:**
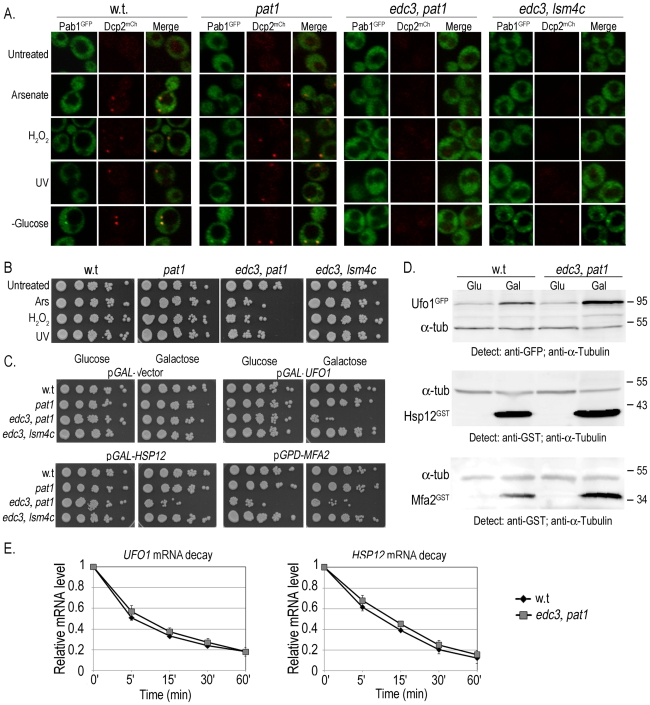
Ectopic high level gene expression affects viability of mutants unable to form PBs and SGs. A. Visualization of wild type, *pat1Δ*, *edc3Δ*, *pat1Δ* or *edc3Δ*, *lsm4Δc* cells expressing the PB marker Dcp2^mCherry^ and the SG marker Pab1^GFP^ untreated or exposed for 30 minutes to 1 mM arsenate or 8.8 mM H_2_O_2_, irradiated with 40 mJ/cm^2^ UV, or incubated in SC medium without glucose for 30 minutes. B. Viability of wild type, *pat1Δ; edc3Δ*, *pat1Δ* or *edc3Δ*, *lsm4Δc* cells untreated (Unt) or treated with 1 mM arsenate, 8.8 mM H_2_O_2_ or irradiated with 40 mJ/cm^2^ UV analyzed by the spot test viability assay. C. Wild type, *pat1Δ; edc3Δ*, *pat1Δ* or *edc3Δ*, *lsm4Δc* cells expressing empty p*GAL*-vector (YCp), p*GAL-GFP-UFO1*, p*GAL-HSP12* or *MFA2*-U1A (pRP1193) were grown in SC medium with 2% glucose or induced in 2% galactose medium overnight, diluted and regrown in the same media to *A*_600_ = 0.5 for spot test analysis on SC plates with 2% glucose or 2% galactose, respectively. D. WB analysis of w.t or *edc3Δ*, *pat1Δ* cells expressing *UFO1*, *HSP12* or *MFA2* from the *GAL* promoter. Glu (noninducing conditions) and Gal (inducing). The intensities of each protein band were normalized to the α-tubulin loading control using ImageJ [79]. E. Comparison of *UFO1* and *HSP12* mRNA decay. p*GAL-GFP-UFO1* or p*GAL-HSP12* was expressed in w.t or *edc3Δ*, *pat1Δ* cells by overnight induction with 2% galactose. Next morning cells at *A*_600_ = 0.5 were washed and transferred to SC medium with 4% glucose. Samples were collected immediately after addition of glucose and at the times indicated and analyzed by qRT-PCR. mRNA levels were normalized to *ACT1* and to time 0 (untreated log cells).

To determine what role sequestration of *UFO1* and of *MFA2* mRNAs in PBs may have we examined the viability of cells defective in the formation of PBs under two different conditions: (a) exposure to arsenate, H_2_O_2_, UV, or glucose deprivation, and (b) high level expression of *UFO1* or *HSP12* from the *GAL* promoter, and of *MFA2* from the strong *GPD* promoter. We used a single *pat1Δ* mutant defective in a mRNA decapping enzyme and two different double mutants: *edc3Δ*, *pat1Δ* mutants defective in both PB and SG formation, and *edc3Δ*, *lsm4Δc* mutants that have reduced PB and SG formation. (Edc3 enhances mRNA decapping [51] and Lsm4 is a subunit of a heptomeric complex involved in mRNA decay [27]). The *edc3Δ* and *lsm4Δ* single mutants are able to form PBs under conditions of glucose deprivation, however, in the absence of Edc3, there is a requirement for the C-terminal prionlike domain of Lsm4 for PB formation [52]. To visualize granule formation isogenic wild type, *pat1Δ*, *edc3Δ*, *pat1Δ*, and *edc3Δ*, *lsm4Δc* cells were transformed to express the PB marker, Dcp2^mCherry^, and the SG marker protein, Pab1^GFP^ from pRP1658. There were no granules in untreated cells in the wild type or in the single *pat1Δ* mutant, PBs appeared in response to arsenate, H_2_O_2_, and UV, and both PBs and SGs appeared after glucose deprivation. No granules were visible in *edc3Δ*, *pat1Δ* mutants consistent with [27]; in response to glucose deprivation the *edc3Δ*, *lsm4Δc* mutants showed very faint PBs and SGs (Figure 8A). Arsenate, H_2_O_2_, and UV treatments caused a decrease in viability of the *edc3Δ*, *pat1Δ* mutants but not of the w.t., *pat1Δ*, or *edc3Δ*, *lsm4Δc* mutants (Figure 8B). Induction of the YCp*GAL* control vector on galactose plates did not affect viability of any of the mutants. In contrast, induction of high levels of *UFO1* or *HSP12* mRNA from the *GAL1* promoter led to a strong decrease in the viability of *edc3Δ*, *pat1Δ* but not of the *pat1Δ*, or *edc3Δ*, *lsm4Δc* mutants compared with wild type or with the noninduced cells growing on glucose plates. *MFA2* is expressed from the constitutive *GPD* promoter and affected viability of these mutants on both glucose and galactose plates (Figure 8C). To determine whether the differential susceptibility of the *edc3Δ*, *pat1Δ* mutants may be the result of non-regulated translation of the *UFO1*, *HSP12* or *MFA2* mRNAs we compared the level of protein in wild type and mutant cells when these three genes were expressed from the *GAL* promoter. Western blot analysis of wild type and *edc3Δ*, *pat1Δ* mutants growing under noninducing (glucose) or inducing (galactose) conditions indicated enhanced translation of each mRNA in the *edc3Δ*, *pat1Δ* mutants (Figure 8D and Figure S4). There was no difference in the rate of decay of *UFO1* or of *HSP12* mRNAs between wild type and *edc3Δ*, *pat1Δ* mutants (Figure 8E).

## Discussion

We analyzed the functional consequence of accumulation of high levels of mRNA in yeast cells under different stress conditions and identified stations in the lifecycle of mRNAs that could explain the discrepancy between transcript abundance and protein level. Our data suggest that the ability to store highly abundant mRNAs in PBs for future regulation is a key facet of the stress response as it allows individual cells to sort mRNAs for decay or translation. A prototype of this kind of regulation is induction of *UFO1* mRNA under stress conditions. Our qRT-PCR experiments show that steady state levels of *UFO1* are elevated in cells stressed by arsenate, H_2_O_2_, or UV. This observation is compatible with microarray data [8], [17], [53]. The Yap1 and Pdr1 dependence of *UFO1* transcription suggests that *UFO1* is part of the early stress response that includes genes that encode several heat shock proteins [54]. Transcriptome studies have shown that production and degradation of mRNA are often coordinated [18]. However, in our experiments even though the steady state *UFO1* mRNA level was elevated after each stress, the mRNA lifecycle was different for UV, an acute stress, compared with arsenate and H_2_O_2_ treatments. Arsenate did not affect *UFO1* mRNA stability, and after H_2_O_2_ there was a very slight destabilization of the mRNA. In contrast UV irradiation led to an elevation of the steady state *UFO1* mRNA level that remained high due to stabilization of the mRNA. In contrast to *UFO1* mRNA there was no marked change in the stability of *HSP12* mRNA after arsenate, H_2_O_2_, or UV. A general stabilization of mRNAs expressed from the *GAL* promoter in response to UV was reported by [55] who did not observe mRNA stabilization after starvation, heat or osmotic stress. UV irradiation did not lead to disassembly of polysomes, however, the mRNAs were no longer associated with the polysomes but accumulated in cytoplasmic granules without losing their polyA tails suggesting they were not designated for decay [55].

By increasing the yeast extract concentration tenfold we were able for the first time to detect Ufo1 protein expressed from genomic *UFO1* after arsenate, H_2_O_2_, and UV. The protein steady state level was very low in untreated cells (1% that of α-tubulin) but after these stresses showed a 10-fold elevation. Examination of the Ufo1 protein half-life after arsenate and H_2_O_2_ showed that the protein was stabilized thus the higher level can be attributed to lack of proteasomal degradation. In contrast UV irradiation did not affect the half-life of Ufo1 protein and in light of Galliard's observation that polysomes are not disassembled after UV irradiation, our interpretation is that the elevated steady state level of Ufo1 protein is due to renewed translation of the stabilized *UFO1* mRNA. This is compatible with the proposed role for Ufo1 in maintenance of genome stability [9] and may be indicative of a remodeling of the genome during recovery. These steady state Ufo1 protein values are comparable to results of a study in which we expressed the bacterial reporter genes, *luxA* and *luxB*, in yeast from the *UFO1* promoter [46]. Luciferase activity was elevated 10-fold in response to 40 mJ/cm^2^ UV whereas arsenate or H_2_O_2_ led to a threefold elevation of enzyme activity. Direct measurement of *UFO1* transcription here by qRT-PCR shows that all three stress treatments lead to very similar elevation of transcription suggesting that luciferase activity may have been affected by prolonged treatment with arsenate or H_2_O_2_. *HSP12* represents a different paradigm of regulation in that the basic steady state mRNA level was higher in unstressed cells and fold-elevation of steady state level was less than for *UFO1*. However, the salient difference was in the cellular amounts of each protein in untreated cells: steady state Hsp12 protein levels are two orders of magnitude those of Ufo1 (Figure S3) and [47]. This may be due to differences in translatability of *HSP12* mRNA that encodes a protein of 107 amino acids compared with *UFO1* whose gene product has 668 residues [56]. Ribosome profiling has shown that translation efficiency can differ 100-fold between different genes with shorter genes having a higher ribosome density [22]. Nuclear export is an important regulatory step for molecular chaperones [57]; in addition the 5′-untranslated region (5′-UTR) plays an important role in determining translation efficiency of *HSP12* mRNA in *Aspergillus oryzae* [58], [59] and Arabidopsis [60].

Cells with the *UFO1*-*MS2L* reporter showed granules after stress; these granules correspond to *UFO1-MS2L* mRNA molecules bound to the fluorescent capsid protein, CP^GFP^. Granules were only visible when the genomic *UFO1* ORF was fused to *MS2L* excluding the possibility they were comprised of aggregated CP^GFP^. Moreover, after cessation of arsenate stress, the granules disappeared; this could be indicative of decay or return to the polysomes. There was a similar decrease of cells with granules after H_2_O_2_ treatment that could be due to cellular responses to reactive oxygen species [3]. *UFO1*-*MS2L* granules were not visible in *UFO1*-*MS2L*, *yap1Δ* or *pdr1Δ* mutants that lack the transcription factors shown by our qRT-PCR results to be essential for induction of *UFO1* by stress. Therefore the granules are indeed *UFO1*-*MS2L* mRNA bound to CP^GFP^. Individual cells showed considerable variation both in their response time and in their number of granules as observed in many other systems. Genes involved in the stress response are expressed with a high level of cell-to-cell variation, stochastic noise attributed to epigenetic factors [61]–[64]. This is considered to enhance the ability of the population to survive adverse conditions by enabling them to sample multiple phenotypes [16], [62].

Colocalization of the *UFO1-MS2L* mRNA granules with the PB and SG marker proteins, Dcp1^GFP^ and eIF4E^RFP^, respectively, indicated that the *UFO1-MS2L* mRNA is sequestered in PBs and SGs. SGs were only visible in the absence of glucose [31] and this necessitated incubation of the cells in SC medium without glucose prior to stress treatment in these experiments. The mRNAs of two highly expressed genes - *UFO1-MS2L* induced by arsenate stress, and *MFA2-U1A* constitutively expressed from the strong GPD promoter - colocalized to the same PBs indicating that these granules house multiple copies of diverse mRNAs. Using protein markers for PBs and SGs we found that these two bodies colocalize after stress consistent with a role for PBs as a sorting station for future regulation of mRNAs for decay or storage [28], [65].

Our data indicate clear differences in the lifecycles of *UFO1* and *HSP12* mRNAs. *UFO1* transcription was elevated three- to fourfold in response to arsenate, H_2_O_2_, or UV and the *UFO1* mRNA was present in PBs and SGs. In contrast, *HSP12* mRNA levels that were double those of *UFO1* mRNA in untreated cells were elevated at most twofold after these stresses. Furthermore under these conditions in which we and [40] observed strong induction of the Hsp12 protein we did not observe granules corrresponding to *HSP12-MS2L* mRNA. The half-life of *UFO1* mRNA was not affected by arsenate and H_2_O_2_ stresses, but the mRNA was stabilized after UV irradiation; none of these stress treatments affected the stability of *HSP12* mRNA. On the protein level we could only observe Ufo1^GFP^ protein in cells with genomic *UFO1-GFP* after stress by taking 10-fold the number of cells for analysis; in contrast genomic Hsp12^GFP^ protein was easily detectable in untreated cells and was elevated between two- and fivefold in cells treated with H_2_O_2_, 37°C, and NaCl using our standard experimental protocols.

Our genetic analysis suggests that sequestration of highly expressed mRNAs in PBs is an important mechanism for survival. In *edc3Δ*, *pat1Δ* mutants that are unable to form PBs we observe elevated protein levels compared with the isogenic wild type cells. mRNA decay rates are not affected in these mutants and our interpretation is that the mRNAs lose their regulation and enter the polysome fraction. Here again we observe a difference between the PB-associated *UFO1* and *MFA2* mRNAs and *HSP12* mRNA: *UFO1* and *MFA2* mRNAs show a six- to eightfold elevation of steady state protein levels, respectively, in the *edc3*Δ, *pat1Δ* mutants compared with *Hsp12* that shows a threefold elevation of protein level compared with wild type cells. Overexpression of all three genes affects the viability of the *edc3*Δ, *pat1Δ* mutants. Pat1 and Dhh1 function in the coordination of translation and PB formation acting as repressors of translation and enhancers of PB formation [30], [35]. The nonregulated elevated translation we observe in the *edc3*Δ, *pat1Δ* mutants may exert its effect on viability through depletion of translation factors required for maintenance of basic essential cell functions. Fold-elevation of *HSP12* mRNA levels in response to stress is lower than that of *UFO1* and the high levels of *HSP12* mRNA produced from the *GAL* promoter may create a requirement for their sequestration in PBs to maintain the balance between storage, decay, and translation. In addition the high level of *HSP12* mRNA could lead to a depletion of essential *HSP*-gene specific regulatory factors and affect biosynthesis of molecular chaperones crucial for withstanding the stress [66], [67].

It is not clear whether the lifecycle of every mRNA species involves a sojourn in PBs. Transcripts of housekeeping genes could go straight to the polysomes [18], [28], or a certain fraction of transcripts, depending on the environmental conditions, could be imprinted with the RNA polymerase II associated Rpb4-Rpb7 heterodimer for sojourn and future sorting in PBs [18]. This heterodimer escorts mRNA transcripts from the nucleus to the cytoplasm and physically interacts with the PB proteins, Pat1 and Lsm2, prior to being reimported into the nucleus [36], [68]. Besides nuclear export [69], Rpb4 and Rpb7 regulate other stages of the mRNA lifecycle such as exit from PBs [70], and 5′ to 3′ and 3′ to 5′ decay [36], [68]. Moreover even though *ASH1-MS2L* and *OXA1-MS2L* CP^GFP^ granules did not show the full colocalization observed for *UFO1* and *MFA2* mRNAs we did observe docking of their granules with PBs, similar to the dynamic docking interaction reported for dendritically localized mRNAs in *Drosophila* neurons [71]. Transport granules share some factors with PBs and this may allow reciprocal transfer of mRNA and proteins between them [28].

**Figure 9:**
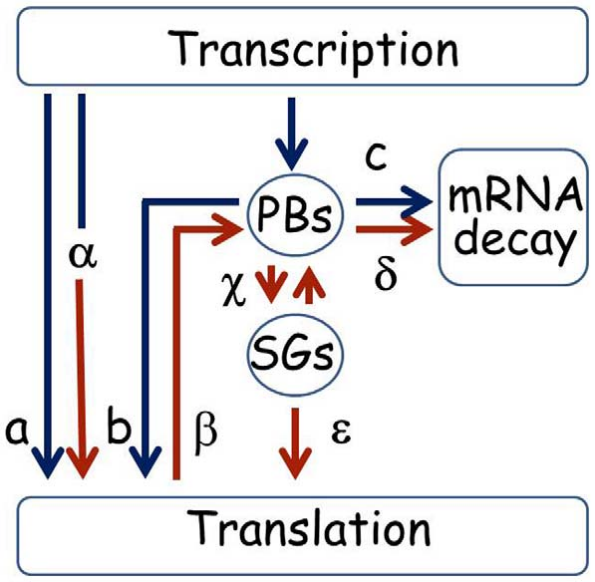
Pathways for mRNA under normal and stress conditions. Under normal growth conditions after transcription a mRNA could go directly to the polysomes for translation (a), or the mRNAs could be escorted by subunits of the RNA polymerase complex to the PBs and sorted either for the polysomes for translation (b), or the mRNA could undergo decay in the PBs (c). Under stress conditions pre-existing mRNAs could undergo enhanced translation as we propose for *HSP12* mRNA (α), or be retracted from the polysomes (β) for future sorting for storage or decay (γ or δ). Furthermore, after stress mRNAs can shuttle between PBs and SGs (γ) from where they can return to translation (ε). mRNA lifecycles under normal growth conditions have blue arrows, stress conditions are indicated with brown arrows. A more comprehensive description of the mRNA lifecycle, particularly of mRNA decay, can be found in [80].

We present a diagram that summarizes putative alternative lifecycles for mRNA based on the sample of genes studied here (Figure 9). The ability to store *UFO1* and other mRNAs in PBs and SGs provides a mechanism for individual cells to regulate their future sorting into pathways for decay or translation. Under normal growth conditions mRNAs could be translated immediately (a), or could pass through PBs that could lead to delayed translation (b); or direction of the mRNA for decay (c). Under stress conditions pre-existing mRNAs undergo enhanced translation as we propose for *HSP12* mRNA (α), or are retracted from the polysomes (β) for future sorting for storage (γ) or decay (δ). Furthermore, after stress mRNAs can shuttle between PBs and SGs (γ) from where they can return to translation (ε). Both extrinsic conditions such as strength and duration of the stress, and the intrinsic metabolic state of a particular cell would influence the outcome [62]–[64]. This regulatory mechanism would result in a population of cells each with the potential to express a unique subset of mRNAs and to acquire a different phenotype thus increasing the ability of the population of genetically identical cells to withstand the stress.

## Materials and Methods

### Yeast strains

Strain genotypes are listed in Table 1. Strain yMK1366 has genomic *DCP1* fused to RFP as a marker for PBs, and strain yMK1307 has genomic *eIF4E* fused to RFP as a marker for SGs [31]. Strain yRP1600 is *pat1Δ*; strain yRP1752 has double deletions of *edc3Δ*, *pat1Δ* [72];and strain yRP2338 has double deletions, *edc3Δ*, *lsm4Δc* [52]. *yap1Δ* or *pdr1Δ* deletion strains were constructed by homologous recombination [73] with a PCR fragment that encodes an auxotrophic *URA3* marker gene amplified from YCp*GAL* [74] flanked with 40 bps for targeting, using the primer pairs yap1::URA3F/R to delete *YAP1*, and pdr1::URA3F/R to delete *PDR1* (Table 2). The deletions were confirmed using the primer pair YAP15′F for *yap1Δ*, and PDR15′F for *pdr1Δ* with the reverse primer URA3midR complementary to the middle of the *URA3* gene.

**Table 1:** Yeast strains.

Yeast strains	Genotype	Reference
BY4741	*MAT* **a**, *his3Δ1, leu2Δ0, met15Δ0, ura3Δ0*	Euroscarf
BY4739	*MATα, leu2Δ0, lys2Δ0, ura3Δ0, YML088w::kanMX4*	Euroscarf
yRP840	*MAT* **a** *his4-539 leu 2-3,112 trp1 ura3-52 cup1::LEU2/PGK1pG/MFA2pG*	(5)
BY4742	*MATα, his3Δ1, leu2Δ0, lys2Δ0, ura3Δ0, YFL014w::kanMX4*	Euroscarf
W303	*MAT* **a**, *ade2, can1, his3, leu2, trp1, ura3*	(9)
UFO1-GFP	*MATα, HIS+, leu2Δ0, met15Δ0, ura3Δ0*	Invitrogen
UFO1-MS2L	*MAT* **a**, *ade2, can1, his3, leu2, trp1, ura3*	This study
UFO1-MS2L	*MATα, ade2, can1, his3, leu2, trp1, ura3*	This study
UFO1-MS2L, yap1Δ	*MATα, ade2, can1, his3, leu2, trp1, ura3, yap1::URA3*	This study
UFO1-MS2L, pdr1Δ	*MATα, ade2, can1, his3, leu2, trp1, ura3, pdr1::URA3*	This study
eIF4E-RFP	*MAT* **a**, *ADE2, his3-11,15, leu2-3, 112,trp1-1, ura3-1, CDC33-RFP::NAT*	(7)
DCP1-RFP	*MATα, ADE2, his3-11, 15,leu2-3, 112,trp1-1, ura3-1, DCP1-RFP:: NAT*	(7)
pat1Δ	*MAT* ***a*** *, his4, leu2, cup1::LEU2PM, trp1, ura3, pat1::LEU2*	(6)
edc3Δ, pat1Δ	*MAT* **a**, *trp1, leu2, ura3, edc3::NEO, pat1::LEU2, cup1::LEU2/PGK1pG/MFA2pG*	(8)
edc3Δ, lsm4Δc	*MAT* **a**, *leu2, trp1, ura3, lys2, his4, cup1::LEU2/PGK1pG/MFA2pG, Lsm4Δc::NEO, edc3::NEO*	(2)
yap1Δ	*MAT* **a**, *ade2, can1, his3, leu2, trp1, ura3, yap1::URA3*	This study
pdr1Δ	*MAT* **a**, *ade2, can1, his3, leu2, trp1, ura3, pdr1::URA3*	This study
HSP12-GFP	*MAT* **a**, *leu2Δ0, met15Δ0, ura3Δ0, GFP(S65T)–His3MX*	(3)
HSP12-MS2L	*MAT* **a**, *ade2, can1, his3, leu2, trp1, ura3*	This study
ASH1- MS2L	*MAT* **a**, *his3Δ1, leu2Δ0, met15Δ0, ura3Δ0, ASH1::loxP::MS2L::ASH13′UTR*	(1)
OXA1- MS2L	*MAT* **a**, *his3Δ1, leu2Δ0, met15Δ0, ura3Δ0, OXA1::loxP::MS2L::OXA13′UTR*	(4)

**Table 2:** Primer list.

yap1::URA3F	GCCACCCAAAACGTTTAAAGAAGGAAAAGTTGTTTCTTAAACCATGTCGAAAGCTACATATAAGGAACGTGCTGC
yap1::URA3R	CATTATAGAAAAAGTTCTTTCGGTTACCCAGTTTTCCATAAAGTTCCCGCTTTAGTTTTGCTGGCCGCATCTTCTCAAATATGC
pdr1::URA3F	CACTTGCTAACTATTCATCTCAGCCAAGAATATACAGAAAAGAATCCAAGAAACTGGAAGATGTCGAAAGCTACATATAAGG
pdr1::URA3R	TTTCGTTGTTGCTAATTCTTTGGTCCTCACTTTGGGTTCCCATCGCTTAAGCAAGTCACTTTAGTTTTGCTGGCCGCATC
YAP1 5′ F	CGGAAACGGCAGTAAACGACG
PDR1 5′F	GCAGGACCATAGCGGCC
URA3midR	CTTCCACCCATGTCTCTTTGAGCAATAAAGCC
UFO1endF	ACAATTGGCCATTGCGTTGTCATTGAGTGAAATCAATTGAACGCTGCAGGTCGACAACCC
UFO13′UTRR	ATAAAATATTTAACATATGCTCTTCCAAATGTACATACTTGCATAGGCCACTAGTGGATC
HSP12endF	GATGCCGTCGAATATGTTTCCGGTCGTGTCCACGGTGAAGAAGACCCAACCAAGAAGTAAACGCTGCAGGTCGACAACCC
HSP123′UTRR	GAATATAATATTAAGGAACATCACACATCATAAAGAAAAAACCATGTAACTACAAAGAGTTCCGAAAGATGCATAGGCCACTAGTGGATC
UFO1ORFF	GCACTCTTAGGATCCCAGGAGGCGCAAGC
HSP12ORFF	GACAACAAGGGTGTCTTCC
HSP123′UTRR	CCACCTCGATTTAAGCG
MS2L-mChF	CCTAAAAGATGGAAACCCGATTCCCTCAGCAATCGCAGCAAACTCCGGCATCTACGGATCCATGGTGAGCAAGGGCGAGG
mCh-GFP2R	GGACGAGCTGTACAAGTAAATCGATACCGTCGACCTCGACATGTCTAAAGGTGAAGAATTATTCACTGGTGTTGTCCCAA
Check-MS2LF	GACTGTTGGTGGTGTAGAGC
Check-mChR	CCGTCCTCGAAGTTCATCAC
UFO1-RTF	TTTGACGGCAGAGTGTTTGG
UFO1-RTR	AACAGACCAGTCCTCTCTTG
HSP12-RTF	CTGAAGCTTTGAAGCCAGAC
HSP12-RTR	TGGAAGACACCCTTGTTGTC
ACT1-RTF	TATGTTCTAGCGCTTGCACC
ACT1-RTR	TAGGAGGTTATGGGAGAGTG

### Fusion of genomic *UFO1* and heat-shock gene, *HSP12*, to the *MS2L* cassette

To the *UFO1* or *HSP12* ORFs we fused a PCR-amplified cassette comprising 12 *MS2L-CP* binding sites and the *Schizosaccharomyces pombe* his5+ selectable marker between two copies of the lambda phage loxP sequence from plasmid p*LoxHis5MS2L* [43]. Forty nts of homology to *UFO1* or *HSP12* were introduced into each primer for site-specific integration of the PCR cassette. The entire *loxP::Sphis5+::loxP::MS2L* PCR cassette was integrated after the STOP codon of *UFO1* using the primers UFO1endF and UFO13′UTRR, and at the end of the *HSP12* ORF using primers HSP12end F and HSP123′UTRR. *UFO1* cassette integration was confirmed by colony PCR amplification using the primer pair, UFO1ORFF/UFO13′UTRR and of *HSP12* with the primer pair, HSP12ORFF/HSP12 3′UTRR. Subsequently the *S. pombe* his5+ marker was excised with Cre recombinase by transforming positive colonies with plasmid p*SH47* that encodes Cre recombinase under the *GAL1* promoter [43]. Transformants were isolated on glucose plates and transferred to galactose medium for excision of the Sphis5+ gene. This yielded histidine auxotrophic colonies in which the *MS2L* sequence was separated from the *UFO1*- or *HSP12* ORF by LoxP followed immediately by the *UFO1* or *HSP12* 3′-UTR. Genomic integration of the *MS2L* cassette downstream of the *UFO1*- and *HSP12* ORF was verified by sequencing the PCR fragment amplified with the primers used to check cassette integration.

### Plasmids

p*DHH1-GFP* [45] and pRP1574 that encodes *EDC3-mCherry* [27] were used as markers of PBs. pRP1658 that encodes both *PAB1-GFP* and *DCP2-mCh*erry was used to provide markers for PBs and SGs [44]. pRP1187 encoding U1A^GFP^ [44] was used to detect *MFA2* mRNA expressed from pRP1193 [24], both are regulated by the *GPD* constitutive promoter. p*CP-MS2L-GFPx3* [43] and p*CP-MS2L-mCherry* (below) were used to detect mRNAs with *MS2L* binding loops; p*GAL-GFP-UFO1* [13], p*GAL-HSP12* [75] and pBM123 (YCp*GAL*) [74] were used in growth analysis studies. p*GAL-GFP-UFO1* [13], p*GAL-HSP12* [75] and p*GAL-MFA2* [75] were used in WB analysis in Figure 8D.

*pCP-MS2L-mCherry plasmid construction*: p*CP-MS2L-mCherry* was constructed by homologous recombination *in vivo* by replacing the first GFP ORF in p*CP-MS2L-GFPx3* with a PCR product that encodes mCherry. The primer pair MS2L-mChF/mCh-GFP2R was designed with homologous ends to *MS2L* and to the third copy of GFP. The reverse primer has a STOP codon after the mCherry ORF so that only mCherry will be expressed fused to the CP. The mCherry PCR product together with p*CP-MS2L-GFPx3* were transformed into wild type yeast for recombination *in vivo*. p*CP-MS2L-mCherry* was extracted from yeast and amplified in *E. coli* for verification of replacement of GFP with mCherry by PCR and sequencing using primer pair Check-MS2LF/Check-mChR. The p*CP-MS2L-mCherry* plasmid was then transformed into *UFO1-MS2L* yeast and treated with arsenate for confirmation of a red signal. Subsequently plasmids pRP1193 [24] and pRP1187 [44] for expression and visualization of *MFA2-U1A* mRNA were cotransformed into the cells for treatment and confocal microscopy.

### Growth conditions

Yeast cultures were grown overnight at 30°C in a rotary thermoshaker at 120 rpm in synthetic minimal medium (SC) supplemented with the appropriate amino acids and carbon source [76]. Next morning cultures were diluted to *A*_600_ = 0.1, regrown to early exponential phase, *A*_600_ = 0.5, and treated as detailed below.

#### Arsenate and H_2_O_2_

For arsenate treatment cells at *A*_600_ = 0.5 in SC glucose medium were incubated with 1 mM Na_2_HAsO_4_; for H_2_O_2_ we diluted the 30% stock solution 1∶1000 to a final concentration of 8.8 mM. Aliquots were collected by brief centrifugation at the times indicated in the figures.

#### UV irradiation

Cells at *A*_600_ = 0.5 were irradiated with 40 mJ/cm^2^ UV on a transilluminator and aliquots were collected as above.

#### Glucose deprivation

Cells at *A*_600_ = 0.5 were collected by brief centrifugation, washed in fresh SC medium without glucose and incubated at 30°C for 30 minutes in fresh SC medium without glucose. Cells were collected by brief centrifugation and visualized by confocal microscopy.

#### Osmotic stress

Cells at *A*_600_ = 0.5 were incubated in 0.5 M NaCl for 30 minutes prior to visualization by confocal microscopy.

#### Heat shock

Cells at *A*_600_ = 0.5 were shifted from 30°C to 37°C for 40 minutes prior to visualization by confocal microscopy.

### Growth analysis

For over-expression experiments YCp*GAL* [74], p*GAL- GFP-UFO1* [13], pRP1193 encoding *MFA2*-U1A [24] or p*GAL-HSP12* [75] were transformed into wild type or mutant yeast. Cells were grown overnight in SC with 2% glucose or induced with 2% galactose, diluted to *A*_600_ = 0.1 in the appropriate media, and regrown to *A*_600_ = 0.5. At this stage viability was assayed using a spot test with 5 µl drops of 10-fold serial dilutions plated on the appropriate medium. Plates were incubated at 30°C for 3 days and scanned.

### Detection of *UFO1-MS2L* or *HSP12-MS2L* mRNA

*UFO1-MS2L* or *HSP12-MS2L* cells were transformed with p*CP-MS2L-GFPx3* that encodes the *MS2L-CP* fused to three tandem repeats of GFP or with p*CP-MS2L-mCherry* (above), both expressed from the inducible *MET25* promoter [42]. Transformed cells at *A*_600_ = 0.5 were incubated in SC medium with 2% glucose but lacking methionine for 1 hour prior to treatment with stress agents as above and visualized by confocal microscopy.

### Quantitative real-time PCR (qRT–PCR)

Total RNA was extracted using a MasterPure Yeast RNA Purification kit (Epicentre) according to the manufacturer's protocol. The amount of total RNA extracted from the cells was measured by absorbance at 260 nm. First-strand cDNA was synthesized from 1 µg total RNA using a blend of RNA primers (random hexamers and anchored oligodT 3∶1 (v/v)) and Verso Enzyme mix following the manufacturer's instructions (Thermo Scientific Verso cDNA Kit). A final concentration of 20 ng/µl of synthesized cDNA was used as template for the PCR reaction. qRT-PCR was performed using Thermo-Start DNA Polymerase, *UFO1* primers (UFO1-RTF and UFO1-RTR), HSP12 primers (HSP12-RTF and HSP12-RTR), or ACT1 primers (ACT1-RTF and ACT1-RTR) and an ABsolute Blue QPCR SYBR Green ROX Mix, as follows: denaturation at 95°C for 30 sec, annealing at 55°C for 30 sec, and extension at 72°C for 30 sec, for 30 cycles. Appropriate non-RT and non-template controls were included in each PCR reaction, and dissociation analysis was performed at the end of each run to confirm the specificity of the reaction. Primer efficiency for *UFO1*, *HSP12* and *ACT1* mRNAs was determined (Figure S5). Data were analyzed using the comparative C_t_ method: [delta][delta]C_t_ = [delta]C_t,sample_−[delta]C_t,reference_. Here [delta]C_T,sample_ is the C_t_ value for each treated sample normalized to the endogenous housekeeping gene Actin and [delta]C_t,reference_ is the C_t_ value for the calibrator (untreated cells) also normalized to Actin (Figure 1). A simple [delta]C_T_ calculation (sample normalized to Actin) was used in Figure 7B.

### mRNA decay

p*GAL-GFP-UFO1* or p*GAL-HSP12* were expressed in *ufo1Δ* or *hsp12Δ* mutants, respectively, by overnight induction with 2% galactose. Next morning cells were diluted to *A*_600_ = 0.1, and regrown to *A*_600_ = 0.5, then treated with the indicated stresses. The cells were washed and transferred to SC with 4% glucose. Samples were collected immediately after glucose addition and at the times indicated and analyzed by qRT-PCR.

### Coexpression of *MS2L*-tagged mRNAs with markers of PB or SGs

Cells expressing *DCP1-RFP* or *eIF4E-RFP* were mated with the *UFO1-MS2L* strain and the diploids were transformed with p*MS2L-CP-GFPx3*. *ASH1-MS2L* or *OXA1-MS2L* cells were transformed with the PB marker plasmid pRP1574 [27] that encodes *EDC3-mCherry*. All strains were treated with stress as described in the relevant figures and visualized by confocal microscopy. To study coexpression of PBs and SGs yeast strains eIF4E-RFP [31] transformed with p*DHH1-GFP* [45] or wild type transformed with pRP1658 [27] were treated as indicated in the relevant figures and visualized by confocal microscopy.

### Microscopy

All images were acquired with an Olympus FV1000 laser-scanning confocal microscope using the ×60 objective lens. The fluorescence was excited with 543 nm for the red fluorescent markers and 488 nm for GFP. For the coexpression experiments sequential screening was used to avoid overlapping. Images are representative of three independent experiments. For the time course experiments images of untreated cells and those exposed to stress (1 mM arsenate, 8.8 mM H_2_O_2_, or 40 mJ/cm^2^ UV) were subjected to quantitative analysis by defining 3 different classes of cells with zero, 1–2, or 3 or more granules. 150–200 cells were assayed for each treatment at each time point. Hoescht staining was used for cell nuclei.

### Induction of protein expression in yeast

For expression from the *GAL1* promoter, yeast cells were grown overnight in 2% galactose medium. GFP-tagged protein was observed using a Nikon fluorescence microscope fitted with GFP-specific filter set: dichromic 505 nm, excitation 450–490 nm, emission (low pass) 515 nm (Nikon).

### Western blot analysis

For Western blot analysis cells were grown overnight in 2% glucose medium, or for induction from the *GAL* promoter with 2% galactose. Next morning the cells were diluted to A_600_ = 0.1 and regrown to A_600_ = 0.5 for TCA precipitation [77] and Western blotting [78].

## Supporting Information

Figure S1 Half-life of ^GFP^Ufo1 protein in w.t. cells in response to stress. ^GFP^Ufo1 protein half-life was determined by expressing p*GAL-GFP-UFO1* in w.t. cells either untreated or exposed to arsenate, H_2_O_2_, UV, or heat shock as described in the Material and Methods. Cells were grown overnight in 2% galactose medium, diluted to *A*_600_ = 0.1 and regrown to *A*_600_ = 0.5. Cycloheximide was added to 10 µg/ml and glucose to 4% at the zero time point and equal aliquots of cells were collected at each time point for TCA precipitation and Western blot analysis. α-tubulin was used as a loading control and the membranes were incubated with anti-GFP and anti-α-tubulin antibodies.
(JPG)

Figure S2 Comparison of Ufo1 and Hsp12 protein levels in wild-type cells. Wild-type cells with GFP-tagged genomic *UFO1* or *HSP12*, untreated or exposed to arsenate, H_2_O_2_, UV, starvation, heat shock, or salt stress. The Ufo1^GFP^ and Hsp12^GFP^ protein levels were normalized to the α-tubulin loading control of the same sample using ImageJ [79] from the anti-GFP Western blots presented in Figure 1C and Figure 7A.
(JPG)

Figure S3 Microscopic analysis of Hsp12^GFP^ protein and of *HSP12-MS2L* mRNA under different stress conditions. A. Cells at *A*_600_ = 0.5 with genomic *HSP12-GFP* for visualization of Hsp12^GFP^ protein were treated for 30 minutes with 1 mM arsenate, 8.8 mM H_2_O_2_, UV-irradiated with 40 mJ/cm^2^, transferred to SC medium without glucose, shifted from 30°C to 37°C, or incubated in 0.5 M NaCl. B. *HSP12-MS2L* cells at *A*_600_ = 0.5 with CP^GFP^ protein for visualization of *HSP12* mRNA were treated with the same stresses as in A.
(JPG)

Figure S4 Relative protein levels in *edc3Δ*, *pat1Δ* mutants compared with wild type. The intensity of the protein bands in the WB in Figure 8D was calculated using ImageJ [79] and normalized to the α-tubulin loading control of the same sample. The normalized values for non-induced (glucose) w.t. and *edc3Δ*, *pat1Δ* mutant and induced (galactose) w.t. and *edc3Δ*, *pat1Δ* mutant are presented in the histogram and accompanying Table. The two right-hand columns indicate the fold induction for each protein in the *edc3Δ*, *pat1Δ* mutant compared with w.t. under noninducing and inducing conditions.
(JPG)

Figure S5 Standard curves for primer pairs used for qRT–PCR. Different amounts of cDNA (1, 50, 100, 150, and 200 ng) prepared as described in the Materials and Methods were assayed in triplicate in qRT-PCR reactions using the primer pairs in Table 2. The results (C_Ts_) were plotted as a function of the log cDNA concentration and the efficiency of each primer pair (E) was calculated using the formula: E = (10^−1/slope−1^)×100. The efficiencies are: *UFO1* = 99.25, *HSP12* = 98.84, *ACT1* = 98.03.
(JPG)
