# Supplementary figures and images for: Sequestration of Highly Expressed mRNAs in Cytoplasmic Granules, P-Bodies, and Stress Granules Enhances Cell Viability
Source: PLoS Genet. 2012 Feb 23;8(2):e1002527. doi: 10.1371/journal.pgen.1002527 (PMC3285586; doi:10.1371/journal.pgen.1002527)

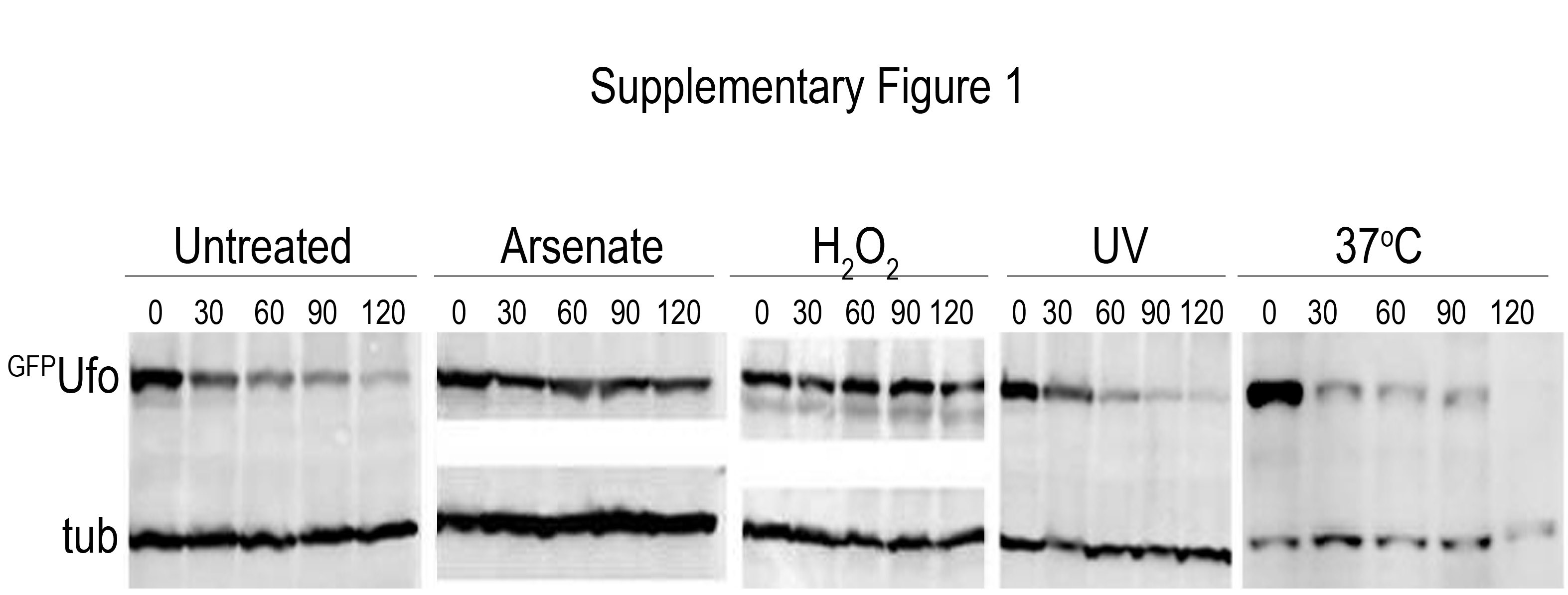

Supplement: Figure S1 — Half-life of GFPUfo1 protein in w.t. cells in response to stress. GFPUfo1 protein half-life was determined by expressing pGAL-GFP-UFO1 in w.t. cells either untreated or exposed to arsenate, H2O2, UV, or heat shock as described in the Material and Methods. Cells were grown overnight in 2% galactose medium, diluted to A 600 = 0.1 and regrown to A 600 = 0.5. Cycloheximide was added to 10 µg/ml and glucose to 4% at the zero time point and equal aliquots of cells were collected at each time point for TCA precipitation and Western blot analysis. α-tubulin was used as a loading control and the membranes were incubated with anti-GFP and anti-α-tubulin antibodies. (JPG) [file pgen.1002527.s001.jpg]

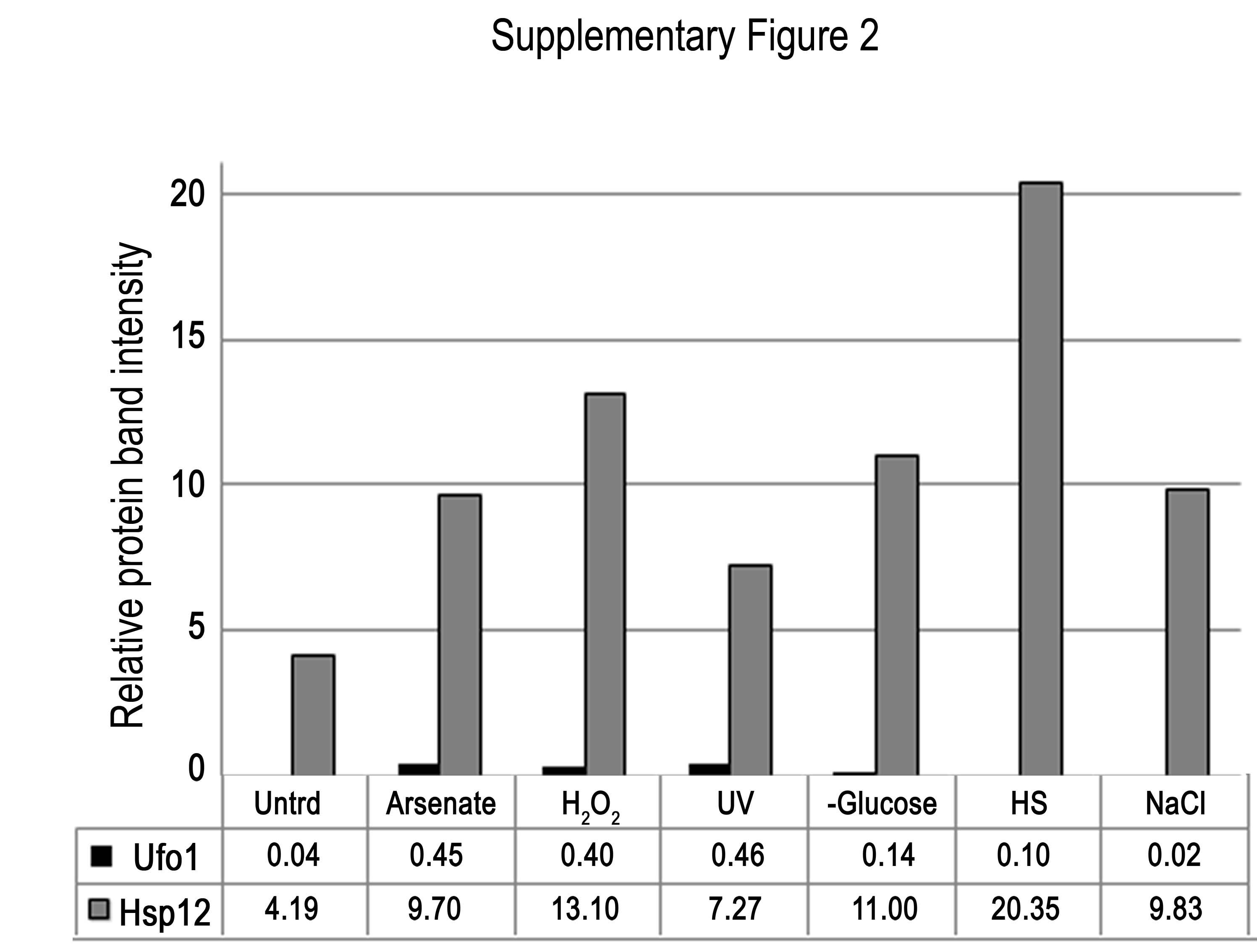

Supplement: Figure S2 — Comparison of Ufo1 and Hsp12 protein levels in wild-type cells. Wild-type cells with GFP-tagged genomic UFO1 or HSP12, untreated or exposed to arsenate, H2O2, UV, starvation, heat shock, or salt stress. The Ufo1GFP and Hsp12GFP protein levels were normalized to the α-tubulin loading control of the same sample using ImageJ [79] from the anti-GFP Western blots presented in Figure 1C and Figure 7A. (JPG) [file pgen.1002527.s002.jpg]

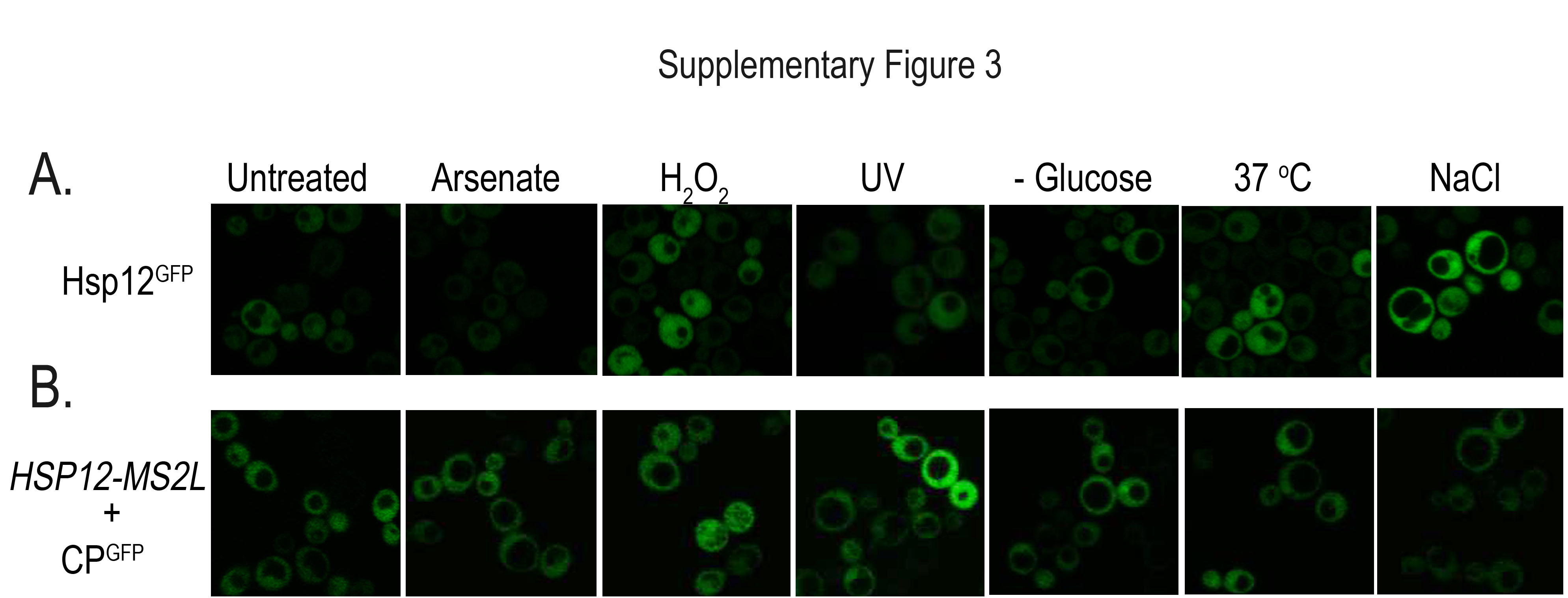

Supplement: Figure S3 — Microscopic analysis of Hsp12GFP protein and of HSP12-MS2L mRNA under different stress conditions. A. Cells at A 600 = 0.5 with genomic HSP12-GFP for visualization of Hsp12GFP protein were treated for 30 minutes with 1 mM arsenate, 8.8 mM H2O2, UV-irradiated with 40 mJ/cm2, transferred to SC medium without glucose, shifted from 30°C to 37°C, or incubated in 0.5 M NaCl. B. HSP12-MS2L cells at A 600 = 0.5 with CPGFP protein for visualization of HSP12 mRNA were treated with the same stresses as in A. (JPG) [file pgen.1002527.s003.jpg]

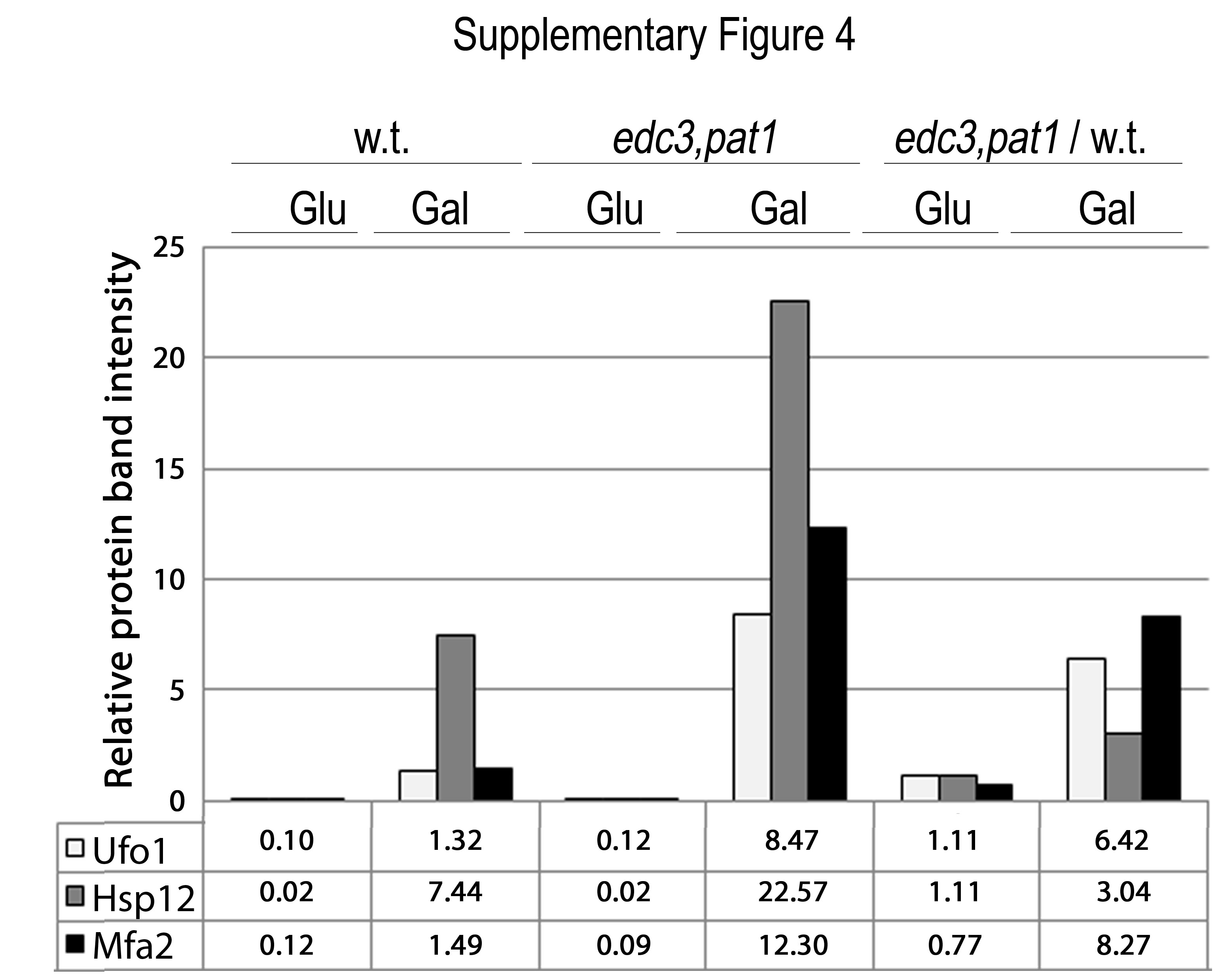

Supplement: Figure S4 — Relative protein levels in edc3Δ, pat1Δ mutants compared with wild type. The intensity of the protein bands in the WB in Figure 8D was calculated using ImageJ [79] and normalized to the α-tubulin loading control of the same sample. The normalized values for non-induced (glucose) w.t. and edc3Δ, pat1Δ mutant and induced (galactose) w.t. and edc3Δ, pat1Δ mutant are presented in the histogram and accompanying Table. The two right-hand columns indicate the fold induction for each protein in the edc3Δ, pat1Δ mutant compared with w.t. under noninducing and inducing conditions. (JPG) [file pgen.1002527.s004.jpg]

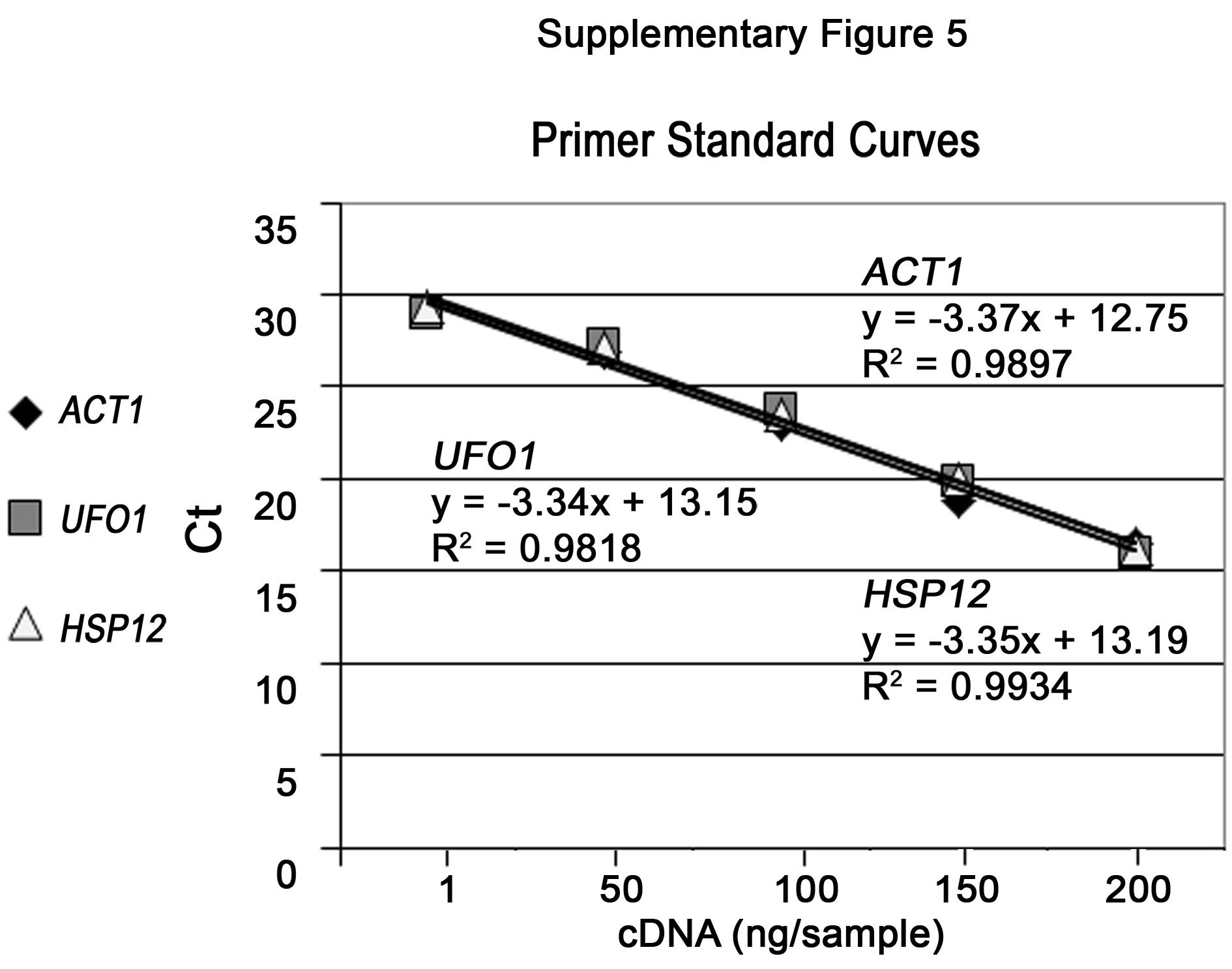

Supplement: Figure S5 — Standard curves for primer pairs used for qRT–PCR. Different amounts of cDNA (1, 50, 100, 150, and 200 ng) prepared as described in the Materials and Methods were assayed in triplicate in qRT-PCR reactions using the primer pairs in Table 2. The results (CTs) were plotted as a function of the log cDNA concentration and the efficiency of each primer pair (E) was calculated using the formula: E = (10−1/slope−1)×100. The efficiencies are: UFO1 = 99.25, HSP12 = 98.84, ACT1 = 98.03. (JPG) [file pgen.1002527.s005.jpg]
